# Management of Primary and Recurrent *Clostridium difficile* Infection: An Update

**DOI:** 10.3390/antibiotics7030054

**Published:** 2018-06-30

**Authors:** Jocelyn Chai, Christine H. Lee

**Affiliations:** 1 University of British Columbia Medical School, Vancouver, BC V6T 1Z3, Canada; Jocelyn.Chai@VIHA.ca; 2 Vancouver Island Health Authority, Victoria, BC V8R 1J8, Canada; 3 Department of Pathology and Molecular Medicine, McMaster University, St Joseph’s Healthcare, Hamilton, ON L8S 4K1, Canada; 4 Department of Pathology and Laboratory Medicine, University of British Columbia, Vancouver, BC V6T 1Z2, Canada

**Keywords:** *Clostridium difficile* infection, healthcare-associated infections, antibiotics, fecal microbiota transplantation

## Abstract

Background: *Clostridium difficile* infection (CDI) is one of the most common healthcare-associated infections (HAI) in the United States and Canada, and incidence rates have increased worldwide in recent decades. Currently, antibiotics are the mainstay treatments for both primary and recurrent CDI, but their efficacy is limited, prompting further therapies to be developed. *Aim:* This review summarizes current and emerging therapies in CDI management including antibiotics, fecal microbiota transplantation, monoclonal antibodies, spore-based therapies, and vaccinations.

## 1. Introduction

*Clostridium difficile* infection (CDI) incidence rates have increased worldwide in recent decades. CDI is the most common healthcare-associated infection (HAI) in the United States (US) [1] and costs an estimated $4.8 billion in acute care facilities alone [2]. This figure does not include the increasing incidence of community-acquired CDI, which has nearly doubled in the past decade [3]. The incidence of multiple recurrent CDI has increased by 188.8% between 2001 and 2012 [4]. Antibiotics are mainstay treatments for both primary and recurrent CDI, with a recent trend toward vancomycin and fidaxomicin over metronidazole [5]. Given the limited efficacy of these antibiotics [6], however, further therapies have been pursued. These include fecal microbiota transplantation (FMT), monoclonal antibodies, newer antibiotics, spore-based therapies, and vaccinations. This article updates our 2015 article and highlights key changes in CDI management [7].

## 2. Vancomycin

Vancomycin is a glycopeptide antibiotic that requires oral ingestion to exert bacteriostatic effects against *C. difficile* via inhibition of bacterial cell wall synthesis [8]. It has long been a standard of care for both primary and recurrent CDI, and the Infectious Diseases Society of America (IDSA) now recommends vancomycin or fidaxomicin over metronidazole for primary and recurrent CDI [5]. This change is based on two large, multicentre randomized controlled trials (RCT) that investigated the clinical success of vancomycin 125 mg four times daily (81.1%; *n* = 259), metronidazole 250 mg four times daily (72.7%; *n* = 278), and tolevamer (44.2%; *n* = 534) (*p* = 0.02) in CDI [9]. Vancomycin was statistically superior to metronidazole in mild, moderate, and severe CDI, with more notable superiority in patients with severe disease (78.5% vs. 66.3%), although this finding was not statistically significant (*p* = 0.059). Both studies also reported fewer CDI recurrences for patients treated with vancomycin, but these findings were not statistically significant. Superiority for vancomycin was previously limited to severe CDI based on an older RCT (*n* = 172) [10].

The recommended dosing regimen of vancomycin depends on the number of recurrences. For an initial nonsevere (WBC ≤ 15,000 cells/mL and serum creatinine < 1.5 mg/dL) or severe CDI episode (WBC ≥ 15,000 cells/mL and serum creatinine > 1.5 mg/dL), vancomycin 125 mg four times daily for 10 days is recommended. However, fulminant CDI may require up to 2 g per day with intravenous metronidazole. Further recurrences require pulsed and tapered vancomycin, which was found in one study to result in fewer recurrences compared to the standard 10-day regimen [11]. (The definitions of severe and complicated/fulminant CDI vary between guidelines, and the above definitions are based on IDSA guidelines [5,12,13]).

## 3. Fidaxomicin

Fidaxomicin is a macrocyclic lactone antibiotic that exerts its bactericidal effect against *C. difficile* via inhibition of bacterial RNA polymerase [14]. Its first-line treatment of primary and recurrent nonfulminant CDI is supported by two double-blinded RCTs (*n* = 1164) comparing fidaxomicin 200 mg twice daily to vancomycin 125 mg four times daily for 10 days [15]. A meta-analysis of these two studies demonstrated noninferiority of fidaxomicin in clinical cure rates compared to vancomycin, although—based on an intention-to-treat (ITT) analysis—fidaxomicin may have improved efficacy in reducing persistent diarrhea or death compared to vancomycin (37% reduction; 95% CI, 2–60; *p* = 0.037). However, modified ITT (mITT) and per-protocol analysis for this finding was not statistically significant [15]. Fidaxomicin was also found to be superior for reducing recurrence rates, persistent diarrhea, and death at day 40 by 40% (95% CI, 26–51; *p* < 0.0001) compared to vancomycin. Fidaxomicin has bactericidal effects and prolonged postantibiotic efficacy compared to vancomycin’s bacteriostatic effects [16].

## 4. Metronidazole

Oral metronidazole has been relegated to alternative therapy in primary, nonsevere CDI (WBC ≤ 15,000 cells/mL and serum creatinine < 1.5 mg/dL) if vancomycin or fidaxomicin are contraindicated or unavailable. However, it is still recommended as an intravenous antibiotic in fulminant CDI (hypotension or shock, ileus, megacolon) as an adjunctive therapy to oral or rectal vancomycin, especially in setting of ileus [17]. Metronidazole can be neurotoxic, potentially causing cerebellar syndrome, encephalopathy, or peripheral neuropathy, especially when administered chronically over weeks to months [18,19]. Therefore, it should be limited to primary CDI treatment until other agents can be used [5].

## 5. Surotomycin

Surotomycin is a novel, oral lipopeptide antibiotic that has demonstrated bactericidal effects in vitro against *C. difficile* [20,21]. An initial, double-blinded Phase 2 RCT (*n* = 209) showed clinical cure rates of surotomycin 125 mg twice daily (92.4%), surotomycin 250 mg twice daily (86.6%), and vancomycin 125 mg four times daily (89.4%) for 10 days. The recurrences were also lower for surotomycin (17.2% for 250 mg; 27.9% for 125 mg) compared to vancomycin (35.6%) [22]. This trial was followed by two parallel, double-blinded Phase 3 RCTs, one of which (*n* = 577) demonstrated that the clinical cure rate of surotomycin 250 mg twice daily (83.4%) was noninferior to vancomycin 125 mg four times daily (82.1%) at Day 10 [23]. In contrast, the parallel Phase 3 study (*n* = 570) failed to show noninferiority at the same doses [24]. In addition, neither study met the criteria for superiority of surotomycin over vancomycin, although recurrence rates were found to be lower for surotomycin compared to vancomycin (14.0% vs. 50.4%; 95% CI, −54.1 to −15.1) [23]. In all three studies, adverse events were similar among treated groups. Further studies are needed to characterize the role of surotomycin.

## 6. Ridinilazole (SMT 19969)

Ridinilazole is a novel, oral antibiotic with minimal systemic absorption that exerts bactericidal effects and reduces both toxin production and cell division of *C. difficile* [25]. A double-blinded, Phase 2 RCT (*n* = 100) showed it to be statistically superior to vancomycin for sustained clinical response rates 30 days after the end of treatment (66.7% vs. 42.4%; *p* = 0.0004) [26] and noninferior to vancomycin for initial cure rate at end of treatment (77.8% vs. 69.7%; 90% CI, −9.3 to 25.8). Adverse events were similar between the two groups. These promising results will need to be corroborated with further trials.

## 7. Fecal Microbiota Transplantation

Fecal microbiota transplantation (FMT) is a type of bacteriotherapy that introduces normal gut bacterial microbes from healthy, screened donors into recipients with dysregulated gut microbes in order to re-establish protective gut flora [27]. FMT has consistently demonstrated efficacy rates of 80 to 90% for clinical remission of recurrent CDI, regardless of the route of administration (such as nasoduodenal, colonoscopy, and enema) [28,29,30,31]. The first RCT of FMT for recurrent CDI patients in 2013 demonstrated significant superiority of a single FMT treatment via nasoduodenal route (81%) over vancomycin (31%) and catalyzed further research. Four more RCTs have investigated the efficacy of FMT vs. vancomycin, placebo, or autologous stool. One reported 90% clinical resolution rates with FMT via colonoscopy compared to 26% with vancomycin, paralleling findings in the initial RCT [29]. Other RCTs showed enema FMT vs. placebo (88.6% vs. 45.5%) and colonoscopy FMT vs. autologous stool (90.9% vs. 62.5%), although the clinical cure rates were variable among the 2 sites in the latter study [30,31]. In contrast, a small RCT (*n* = 30) comparing vancomycin followed by enema FMT and tapered vancomycin found comparable efficacy (43.8% vs. 58.3%) between the groups [32]. This finding is likely attributable to a single administration of FMT, increased efficacy of tapered vancomycin over regimens in previous FMT trials, and possible residual vancomycin affecting FMT efficacy, and the small number of patients. While many studies use multiple administrations of FMT in determining efficacy rates, single administrations of FMT may have lower CDI resolution rates with reported values of 62%, 65%, and 70% in 3 different RCTs [29,33,34]. A recent meta-analysis of these 5 RCTs (*n* = 284) reported that FMT was significantly more effective than its comparators (vancomycin, placebo, autologous stool) in providing a clinical cure of CDI with an RR 0.41 (95% CI, 0.22–0.74; *p* = 0.004) and had an NNT of 3 (95% CI, 2–7), although heterogeneity was noted and attributed to the location of the trial (Europe vs. North America) and the route of administration [35].

The best route of FMT administration has not been established. The previous meta-analysis determined that nasoduodenal and colonoscopy may be more effective than enema [35]. However, another meta-analysis determined that the clinical resolution rate was higher for lower gastrointestinal (GI) delivery including colonoscopy/enema (91.2%) compared to upper GI delivery (80.6%) including nasogastric/nasojejunal/gastroscopy [36]. One RCT (*n* = 20) showed not statistically significant differences between nasogastric and colonoscopy (60% vs. 80%; *p* = 0.628), while another (*n* = 116) randomized FMT by oral capsule or colonoscopy and found comparable clinical cure rates (96.2% vs. 96.2%, *p* < 0.001) [34,37]. Further studies are needed to characterize the best route, particularly for oral-encapsulated FMT, which would allow for commercialization and more widespread use of FMT therapy. The preparation of FMT—whether fresh, frozen, or lyophilized—can also expand use. Frozen FMT has been found to be noninferior to fresh FMT in 2 RCTs [33,38], and is more convenient and cost-effective. However, lyophilized FMT was found to be inferior to fresh in one small RCT [38].

The adverse effects of FMT are limited to postprocedural symptoms that resolve within a few hours and include bloating, abdominal pain, flatulence, diarrhea, and constipation [39]. Transmission of pathogens from donor to recipient has not been found in the literature beyond 2 cases of norovirus infection [40]. Long-term efficacy data on FMT is sparse, although there have been reports describing the development of autoimmune diseases (Sjögren’s syndrome, rheumatoid arthritis, idiopathic thrombocytopenia), GI disorders (ulcerative colitis flares, microscopic colitis), peripheral neuropathy, and weightgain following FMT [41,42,43]. In comparison to vancomycin and placebo treatments, FMT did not differ in number of serious adverse events (death, hospitalizations) with an RR 0.64 (95% CI, 0.26–1.61) [35].

Guidelines on CDI management have been updated to include FMT. The Infectious Disease Society of America (IDSA) recommends using FMT after antibiotic failure for at least two CDI recurrences (three total episodes) [5]. The American College of Gastroenterology recommends at least three CDI recurrences (four total episodes) following a pulsed vancomycin treatment course [13]. The European Society of Clinical Microbiology and Infectious Disease (ECSMID) Guidelines recommend FMT after “multiple, recurrent CDI unresponsive to repeated antibiotic treatment” and in combination with oral antibiotics [12]. It remains unclear when FMT should be administered during recurrence. The delay between first onset of CDI and FMT treatment and its use in combination with antibiotics can be explained by how the treatment has been studied in clinical trials. Current barriers to FMT use include aesthetics, the invasive nature of delivery, and the need for personnel to administer it. The advent of oral-encapsulated FMT would remove these barriers.

## 8. Spore-Based Therapy

Nontoxigenic *Clostridium difficile* (NTCD) spore, M3, is derived from the bacterium that does not possess the genes for toxin production [44]. A double-blinded, Phase 2 RCT (*n* = 168) showed that it reduced recurrences of CDI from 30% with placebo to 11% with NTCD-M3 (OR 0.28; 95% CI, 0.11–0.69; *p* = 0.006) [44]. Colonization of NTCD-M3 was initially achieved in 69% of patients; however, after week 22, this strain was not detected.

SER-109 is another spore-based therapy comprising of around 50 species of Firmicutes spores isolated from healthy stool donors [45]. An open-label study (*n* = 30) reported efficacy of 86.7% in clinical cure rates for up to 8 weeks [45]. Colonization with spores was detected at week 24. A Phase III study (ECOSPOR III) on SER-109 vs. placebo is currently being conducted [46]. Further research is required to determine the role for spore-based therapy in CDI management.

## 9. Passive Immunization: Bezlotoxumab and Actoxumab

Bezlotoxumab is a monoclonal antibody for passive immunization against *C. difficile* toxin B by inhibiting the binding of toxin B to host cells [47]. MODIFY I and MODIFY II, two multisite Phase 3 studies including 2655 patients, reported recurrence rates in bezlotoxumab vs. placebo groups of 17% vs. 28% (95% CI, −15.9 to −4.3; *p* < 0.001) and 16% vs. 26% (95% CI, −15.5 to −4.3; *p* < 0.001), respectively [48]. This data has resulted in approval by the FDA in 2016 for its use as an adjunctive to antibiotics in reducing CDI recurrences. Actoxumab is also a monoclonal antibody, but against *C. difficile* toxin A. In combination with bezlotoxumab, it has been shown to further decrease recurrence rates in MODIFY I (16% vs. 28%; *p* < 0.001) and in MODIFY II (15% vs. 26%; *p* < 0.001). At 12 weeks, sustained clinical cure rates were 64% for bezlotoxumab alone, 58% for the combination, and 54% for placebo. It is unclear whether this therapy reduces the severity or duration of CDI symptoms in patients who have recurrences.

## 10. Active Immunization: Vaccines

Vaccines against *C. difficile* have the potential to allow a long-term, adaptive immune response against toxins A and B [49]. A few vaccines are being tested in clinical trials including a Phase 3 trial (Clover; Pfizer) [50], although results have not yet been published. Another Phase 3 trial for *C. difficile* vaccines (Cdiffense; Sanofi Pasteur) has been terminated due to low likelihood of attaining its primary outcome [51]. A phase 2 study (VLA84; Valneva) showed promising results, although Valneva has not yet continued the study [52].

## 11. Conclusions

Recent advances in *C. difficile* management for primary and recurrent CDI have improved current practices. Although antibiotic therapy with vancomycin and fidaxomicin remains first-line, alternative treatments have an important role, especially in treating recurrent CDI. Further research is underway to determine the efficacy of newer antibiotics for primary episodes of CDI and prevention of future recurrences along with vaccines and antibiotic-sparing therapies for CDI management.
